# In vitro and in vivo evaluation of two combined β-lactamase inhibitors against carbapenem-resistant *Acinetobacter baumannii*

**DOI:** 10.1007/s10096-023-04664-z

**Published:** 2023-09-15

**Authors:** Andrea Vila Domínguez, Irene Molina Panadero, Younes Smani

**Affiliations:** 1 Instituto de Biomedicina de Sevilla (IBiS), Hospital Universitario Virgen del Rocío/CSIC, Universidad de Seville, Seville, Spain; 2 Centro Andaluz de Biología del Desarrollo, Universidad Pablo de Olavide/Consejo Superior de Investigaciones Científicas/Junta de Andalucía, Sevilla, Spain; 3 Departamento de Biología Molecular e Ingeniería Bioquímica, Universidad Pablo de Olavide, Sevilla, Spain

**Keywords:** *Acinetobacter baumannii*, Clavulanic acid, Tazobactam, Combination

## Abstract

The objective of this study was to evaluate the in vitro and in vivo efficacy of clavulanic acid (C/A) in combination with tazobactam against clinical strains of carbapenem-resistant *Acinetobacter baumannii*. The MIC of 24 clinical strains of *A. baumannii* was determined, and a checkerboard assay and time-kill curve analysis were performed in selected strains to determine the synergy between C/A and tazobactam. The efficacy of C/A in monotherapy and in combination with tazobactam was evaluated in vitro in cell culture experiments and in a murine peritoneal sepsis model. The C/A and C/A plus tazobactam MIC_50_ were 128 and <1 mg/L, respectively. The checkerboard assay showed that tazobactam (4 and 8 mg/L) demonstrated synergy with C/A against *A. baumannii* Ab40, an OXA-24 producer strain, and Ab293, a lacking OXA β-lactamase strain. The time-kill curve assay showed both bactericidal and synergistic effects against Ab40 and Ab293, with C/A 1xMIC and tazobactam (4 and 8 mg/L) at 24 h. In the murine peritoneal sepsis model with Ab293 strain, the combination of C/A and tazobactam reduced bacterial loads in tissues and blood by 2 and 4 log_10_ CFU/g or mL compared with C/A alone. Combining C/A with tazobactam could be considered as a potential alternative strategy to treat *A. baumannii* in some cases, and future work with more strains is needed to confirm this possibility.

## Introduction

*Acinetobacter baumannii* is a multidrug-resistant (MDR) Gram-negative bacterium that is responsible for a large number of hospital-acquired infections, such as ventilator-associated pneumonia, bloodstream infections, burn and soft tissue infections, meningitis, and osteomyelitis [1]. The first-line therapeutic options for this pathogen have included broad-spectrum β-lactams (BLs), such as carbapenems, for several years [2, 3]. However, *A. baumannii* has developed mechanisms that confer resistance to these antibiotics, including decreased outer membrane permeability, efflux pumps, penicillin-binding protein modification, and the production of β-lactamases [4].

The emergence of MDR *A. baumannii* has made it difficult to find an effective antimicrobial treatment for infections caused by this pathogen. In response, the World Health Organization has designated *A. baumannii* resistant to carbapenems as a critical priority pathogen that poses a significant threat to human health and for which new antibiotics are urgently needed [5].

To overcome the loss of BL antibiotic activity, β-lactamase inhibitors (BLIs) were developed to be used in combination with BLs in order to inhibit β-lactamase and allow BLs to act unhindered [4, 6]. In general, commercial BLIs have low antibiotic activity against *A. baumannii* and are not used as single antimicrobial agents. However, sulbactam has demonstrated good antibacterial activity both in vitro and in animal models against *A. baumannii* [6–8]. This antibacterial activity is mediated through the inhibition of the penicillin-binding proteins (PBPs) PBP1 and PBP3 [9].

Clavulanic acid (C/A), sulbactam, and tazobactam are irreversible “suicide inhibitors” that can permanently inactive β-lactamase through secondary chemical reactions in the enzyme’s active site. These inhibitors have a high affinity for many class A β-lactamases but do not provide protection against class B, C, and D β-lactamases [10–12]. *A. baumannii* carbapenem-hydrolyzing class D β-lactamase (CHDLs) such as OXA-23, OXA-24, and OXA-58 are the main cause of carbapenem resistance, which are recalcitrant to inhibition against most commercially available inhibitors [13].

Although the activity of C/A and tazobactam against *A. baumannii* is lower than that of sulbactam, two studies have reported a range of minimum inhibitory concentrations (MICs) of C/A from 2 to 256 mg/L, with MICs of ≤8 mg/L for 29 and 40.9% of the *A. baumannii* strains tested, respectively [6, 14]. In vivo, C/A has demonstrated therapeutic efficacy against carbapenem-susceptible *A. baumannii* by reducing the bacterial loads in the lungs by 2–2.5 log CFU/g and increasing the frequency of sterile blood cultures [14]. On the other hand, tazobactam has been reported to have antibacterial activity against *A. baumannii,* with a MIC of 16 mg/L in vitro and a 1-log CFU reduction in a murine lung infection model [15]. In a study of 54 MDR *A. baumannii* strains, sulbactam had a MIC range from 16 to 256 mg/L, while tazobactam had a MIC range from 32 to 512 mg/L [16].

The combination of BLIs is being developed for the treatment of *A. baumannii* infections. Studies have shown potent in vitro activity of sulbactam/durlobactam, sulbactam/avibactam, and sulbactam/LN-1-255 against clinical isolates of *A. baumannii* in China, Argentina, and Spain, respectively [17–19] and in vitro and in vivo activity of sulbactam/ETX2514 against carbapenem-resistant *A. baumannii* [20, 21]. Additionally, in vitro and in vivo activity of sulbactam/YTR830H (another β-lactamase inhibitor) has been observed against *A. calcoaceticus* [22]. Durlobactam, avibactam, ETX2514, LN-1-255, and YTR830H significantly increased the susceptibility of clinical isolates of *A. baumannii* to sulbactam. To date, there is no reported data on the susceptibility data for C/A in combination with tazobactam against carbapenem-intermediate and carbapenem-resistant *A. baumannii*. Thus, the main aim of this study was to determine the efficacy of C/A alone and in combination with tazobactam against two selected carbapenem-resistant *A. baumannii* strains.

## Material and methods

### Bacterial strains

A total of 24 clinical strains of carbapenem-intermediate (*n*=4) and carbapenem-resistant (*n*=20) *A. baumannii* were collected from the “II Spanish Study of *A. baumannii* GEIH-REIPI 2000-2010” multicenter study (Genbank Bioproject PRJNA422585) for use in this study. The strains were chosen due to their carbapenem and C/A non-susceptible profiles.

### Antimicrobial agents and in vitro susceptibility testing

Standard laboratory powders of C/A (Sigma, Spain) and tazobactam (Sigma, Spain) were used. The MICs of C/A alone and in combination with tazobactam were determined against 24 clinical strains of carbapenem-intermediate and carbapenem-resistant *A. baumannii* in two independent experiments using the broth microdilution method, in accordance with the standard guidelines of the European Committee on Antimicrobial Susceptibility Testing (EUCAST) [23]. A 5×10^5^ CFU/mL inoculum of each strain was cultured in Mueller Hinton Broth (MHB) and added to U bottom microtiter plates (Deltlab, Spain) containing C/A alone and C/A and 4 mg/L of tazobactam. The plates were incubated for 18 h at 37°C. *A. baumannii* ATCC 17978 was used as a control strain. The MIC_50_ and MIC_90_, which represent the concentrations that were effective against ≥50 and ≥90% of the isolates tested, were determined.

### Checkerboard assay

To determinate the synergistic activity between C/A and tazobactam, two strains of carbapenem-resistant *A. baumannii* (Ab40, an OXA-24 producer strain, and Ab293, a lacking OXA β-lactamase strain) were selected for further studies. The assay was performed in duplicate using a 96-well plate as described previously [24]. C/A (from 0 to 64 mg/L) was serially diluted 2-fold along the x axis, while tazobactam (from 0 to 64 mg/L) was serially diluted 2-fold along the y axis to create a matrix of different combinations of both agents at different concentrations. Bacterial cultures grown overnight were diluted in saline to a 0.5 McFarland turbidity and further diluted 1:50 in MHB before being inoculated into each well to achieve a final concentration of approximately 5.5×10^5^ CFU/mL. The 96-well plates were then incubated at 37 °C for 18 h and examined for visible turbidity. The fractional inhibitory concentration (FIC) of C/A was calculated by dividing the MIC of C/A in the presence of tazobactam by the MIC of C/A alone. Similarly, the FIC of tazobactam was calculated by dividing the MIC of tazobactam in the presence of C/A by the MIC of tazobactam alone. The FIC index (FICI) was the sum of both FIC values. FICI values of ≤0.5 and >0.5 were interpreted as synergistic and additive, respectively.

### Time-kill kinetic assays

In order to determine the bactericidal and synergistic activity, time-kill curves of the Ab40 and Ab293 strains were performed in duplicate as previously described [24]. An initial inoculum of 1×10^6^ CFU/mL was prepared in MHB in the presence of 1xMIC of C/A alone or in combination with 4 and 8 mg/L of tazobactam. A drug-free broth was evaluated in parallel as a control. Tubes of each condition were incubated at 37°C with shaking, and viable counts were determined by serial dilution at 0, 2, 4, 8, and 24 h. Viable counts were determined by plating 100 µL of the control, test cultures, or the respective dilutions at the indicated times onto sheep blood agar plates (Thermo Fisher, Spain). Plates were incubated for 24 h at 37 °C, and after colony counts, the log_10_ of viable cells (CFU/mL) was determined. Synergy was defined as a reduction of ≥ 2 log_10_ CFU/mL with the combination compared to the more active drug [24]. Therefore, tazobactam was considered synergistic when, in combination with C/A, it reduced the bacterial concentration by ≥ 2 log_10_ CFU/mL compared to C/A alone. Bactericidal activity was defined as a reduction of ≥3 log_10_ CFU/mL from the initial inoculum [25].

### Human cell culture

HeLa cells were grown in 24-well plates in DMEM supplemented with 10% heat-inactivated fetal bovine serum (FBS), vancomycin (50 mg/L), gentamicin (20 mg/L), amphotericin B (0.25 mg/L) (Invitrogen, Spain), and 1% HEPES in a humidified incubator with 5% CO_2_ at 37°C. HeLa cells were routinely passaged every 3 or 4 days. Immediately before infection, HeLa cells were washed three times with prewarmed PBS and further incubated in DMEM without FBS and antibiotics [26].

### Adhesion-invasion assays

HeLa cells were infected with 1×10^8^ CFU/mL of *A. baumannii* Ab40 and Ab293 strains in the absence and presence of 1xMIC of C/A alone or in combination with 4 and 8 mg/L of tazobactam at a multiplicity of infection (MOI) of 100 for 2 h with 5% CO_2_ at 37°C. Subsequently, infected HeLa cells were washed five times with prewarmed PBS and lysed with 0.5% Triton X-100. Diluted lysates were plated onto LB agar (Merck, Spain) and incubated at 37°C for 24 h for enumeration of developed colonies and then the determination of the number of bacteria that attached-invaded to HeLa cells [26].

### Animals

Female C57BL/6 mice weighing 18 to 20 g and considered immunocompetent were obtained from the University of Seville. The mice were certified as pathogen-free and genetically authenticated and were housed in regulated cages with access to food and water ad libitum. This study was conducted in accordance with the UK Animals (Scientific Procedures) Act 1986 and associated guidelines, as well as the European Communities Council Directive of 24 November 1986 (86/609/EEC). The animal model of this study was approved by the Committee on the Ethics of Animal Experiments at the University Hospital of Virgen del Rocio in Seville, Spain. All surgeries were performed under sodium thiopental anesthesia, and measures were taken to minimize animal suffering.

### *A. baumannii* peritoneal sepsis models

A murine peritoneal sepsis model caused by the carbapenem-resistant *A. baumannii* Ab40 OXA-24 producer was established by intraperitoneal inoculation of the bacteria in immunocompetent mice [24]. The Minimal Bacterial Lethal Dose 100 (MLD100) was determined by inoculating two groups of 6 mice each with 0.5 mL of the decreasing amounts of bacterial inoculum from 9 to 8 log_10_ CFU/mL and monitoring the survival of the mice for 7 days.

### Therapeutic effect of clavulanic acid in monotherapy and in combination with tazobactam in a murine model of peritoneal sepsis

A murine peritoneal sepsis model was established by intraperitoneal inoculation of mice with the carbapenem-resistant *A. baumannii* Ab40 strain and treated with either C/A monotherapy or C/A combined with tazobactam. The mice were infected with 0.5 mL of the MLD100 of Ab40 strain (9 log CFU/mL) and randomly ascribed to the following groups: (i) controls (no treatment); (ii) C/A administered intraperitoneally at 13 mg/kg/4 h starting 4 h after bacterial inoculation, for 24 h [14]; (iii) C/A administered intraperitoneally at 13 mg/kg/4 h plus tazobactam administered intraperitoneally at 32 mg/kg/4 h starting 4 h after bacterial inoculation, for 24 h [27]. At the end of the experiment, after the mice died or were sacrificed, aseptic thoracotomies were performed, blood samples were obtained by cardiac puncture, and the spleen and lungs were aseptically removed and homogenized (Stomacher 80; Tekmar Co., USA) in 2 mL of sterile 0.9% NaCl solution. Tenfold dilutions of the homogenized spleen and lungs and blood were plated onto sheep blood agar for quantitative cultures.

### Statistical analysis

Group data is presented as the mean ± standard error of the mean (SEM). Differences in bacterial concentrations in the spleen, lung, and blood (mean ± SEM log_10_ CFU/g or log_10_ CFU/mL) were analyzed using analysis of variance (ANOVA) and post hoc Dunnett’s and Tukey’s tests. *P* values less than 0.05 were considered significant. The statistical analysis was performed using SPSS version 21.0 (SPSS Inc.).

## Results

### In vitro activity of clavulanic acid alone and in combination with tazobactam

C/A alone and in combination with tazobactam were tested against 24 clinical strains of carbapenem-intermediate and carbapenem-resistant *A. baumannii*. The results of the MICs tests are displayed in Table 1. The MICs ranged from 16 to >256 mg/L for C/A and from <1 to 16 mg/L for the combination with tazobactam. The control strain ATCC 17978 present an MIC of 32 mg/L for C/A and <1 mg/L for the combination with tazobactam. The MIC_50_ and MIC_90_ concentrations, which represent the concentration effective for 50 and 90% of the isolates tested, respectively, for C/A alone were 128 and >256 mg/L, respectively. However, the MIC_50_ and MIC_90_ for C/A in combination with tazobactam were <1 and 16 mg/L, respectively.

**Table 1:** MIC determination of clavulanic acid alone and in combination with tazobactam against clinical isolates of *A. baumannii*

Strain	IMP	MPM	C/A	C/A + 4 mg/L of TAZ
ATCC 17978*	0.5	0.5	32	<1
Ab16	64	>64	32	<1
Ab17	8	8	64	<1
Ab19	16	8	256	8
Ab37	16	8	16	<1
Ab40	64	>64	16	<1
Ab53	64	16	128	32
Ab286	2	16	32	<1
Ab288	4	32	64	<1
Ab289	2	8	32	<1
Ab293	16	8	32	<1
Ab295	8	4	128	4
Ab298	16	8	128	8
Ab299	16	8	>256	16
Ab303	32	16	128	16
Ab399	64	>64	256	2
Ab405	32	16	64	16
Ab410	32	16	128	<1
Ab414	32	16	64	<1
Ab416	32	16	32	<1
Ab417	16	8	128	<1
Ab440	2	8	256	<1
Ab441	2	8	128	<1
Ab448	8	8	128	1
Ab453	4	4	128	<1

*IMP* imipenem, *MPM* meropenem, *C/A* clavulanic acid, *TAZ* tazobactam

*ATCC 17978: control strain

The checkerboard assay indicated that tazobactam at concentrations of 4 and 8 mg/L had a synergistic effect with C/A against Ab40, an OXA-24 producer strain, and Ab293, a lacking OXA β-lactamase strain. When combined with C/A, tazobactam at 4 and 8 mg/L enhanced the activity of C/A against the Ab40 strain, resulting in an FIC index (FICI) of 0.275. Similarly, the combination of tazobactam at 4 and 8 mg/L with C/A increased the activity of C/A against the Ab93 strain, yielding an FICI of 0.25 (Table 2). In contrast, the combination of tazobactam at lower concentrations, such as 1 and 2 mg/L, with C/A caused additive effects instead of synergy, yielding an FICI > 0.5 for both strains (data not shown). As a result, the optimal concentrations for further experiments were determined to be 4 and 8 mg/L of tazobactam.

**Table 2:** MIC determination of clavulanic acid and tazobactam alone or in combination against carbapenem-resistant *A. baumannii* strains

Strain	MIC mg/L				
	C/A	TAZ	C/A in the presence of 4 mg/L of TAZ	FICI	Fold change in C/A
Ab40	16	16	<1	0.275	>16
Ab293	32	16	<1	0.25	>32

The MICs of combined C/A with tazobactam were equal at 4 or 8 mg/L of tazobactam

*C/A* clavulanic acid, *TAZ* tazobactam, *FICI* fractional inhibitory concentration index

### Time-kill curves

**Fig. 1:**
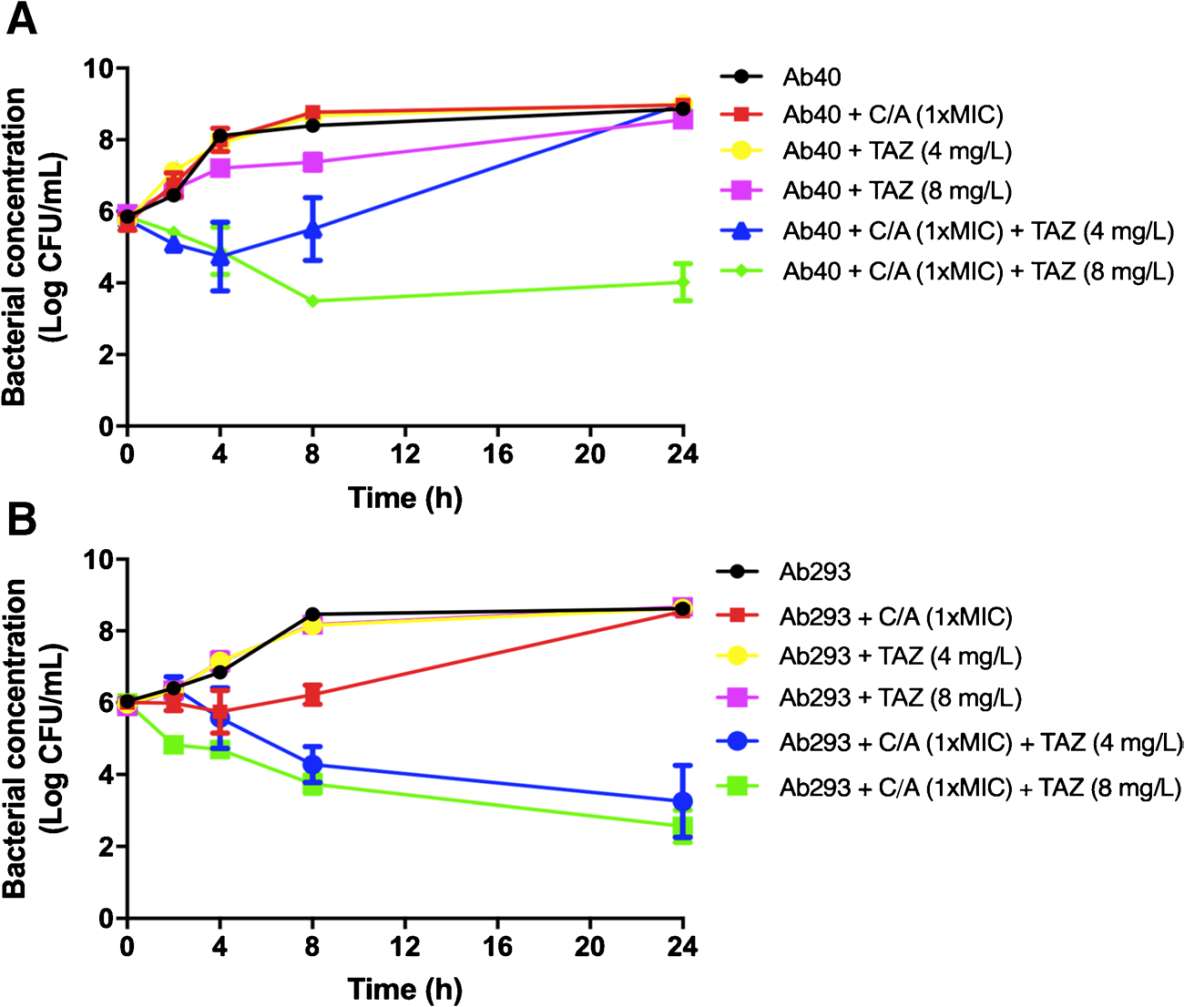
Tazobactam potentiates clavulanic acid activity against two selected carbapenem-resistant *A. baumannii*. Time-kill curves of *A. baumannii* Ab40 OXA-24 producer strain (**A**) and Ab293 strain (**B**) in the presence of 4 and 8 mg/L tazobactam and 1xMIC clavulanic acid, alone or in combination with tazobactam for 24 h. C/A, clavulanic acid; TAZ, tazobactam. Data are represented as mean ± SEM from two independent experiments

Using time-course assays, we evaluated the bactericidal activity of tazobactam in combination with C/A against Ab40 and Ab293 strains. Figure 1A illustrates that the combination of 8 mg/L tazobactam with 16 mg/L C/A (1xMIC for the Ab40 strain) exhibited a synergistic and bactericidal effect after 8 h, reducing the bacterial count by over 3 log_10_ CFU/mL compared to C/A alone. These bactericidal and synergistic effects persisted until 24 h. In contrast, the combination of 4 mg/L tazobactam with 1xMIC C/A only showed synergistic activity at 4 h in comparison to C/A alone. For the Ab293 strain, 4 and 8 mg/L tazobactam in combination with 32 mg/L C/A (1xMIC for Ab293 strain) demonstrated a synergistic effect after 8 and 24 h by reducing the bacterial count by over 2 and 3 log_10_ CFU/mL, respectively, compared to C/A alone. This effect was intensified at 24 h, where the two concentrations of tazobactam combined with C/A showed a bactericidal effect (Fig. 1B).

### Effect of clavulanic acid in combination with tazobactam on the bacterial adherence invasion to host cells

**Fig. 2:**
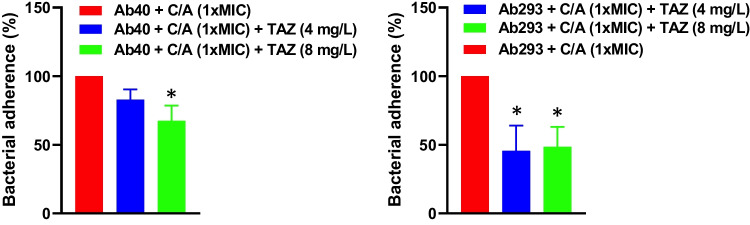
Effect of clavulanic acid alone or in combination with tazobactam on *A. baumannii* interaction with host cells. HeLa cells were pretreated with C/A (1xMIC) without or with tazobactam (4 and 8 mg/L) and infected with carbapenem-resistant *A. baumannii* Ab40 OXA-24 producer and Ab293 strains. The assay of *A. baumannii* adherence invasion to HeLa cells for 2 h was performed as described in “Material and methods.” C/A, clavulanic acid; TAZ, tazobactam. Data are represented as mean ± SEM from three independent experiments. *P < 0.05 *vs*. the clavulanic acid treatment group

To evaluate the effect of C/A in combination with tazobactam in *A. baumannii* interaction with host cells, we studied the adherence invasion of Ab40 and Ab293 strains on HeLa cells for 2 hours in the presence of C/A with or without tazobactam. We showed that treatment with C/A at 1xMIC plus tazobactam at 4 and 8 mg/L reduced the counts of adherent-invasive Ab40 to HeLa cells by 17 and 32% (*P*<0.05), respectively, when compared with C/A monotherapy. Of note, a more enhanced reduction has been observed with the Ab293 strain, for which the two combination treatments reduced its adherent-invasive counts by 54% (*P*<0.05) and 57% (*P*<0.05), respectively, when compared with C/A monotherapy (Fig. 2).

### In vivo activity of clavulanic acid alone and in combination with tazobactam against *A. baumannii* infection

To verify the in vitro synergistic effect of C/A and tazobactam against carbapenem-resistant *A. baumannii* and to study this synergy in a living organism, we conducted an experiment using a vertebrate model of infection by this pathogen. First, we determined the MLD100 of the Ab40 strain. Mice exposed intraperitoneally to 0.5 mL of the Ab40 strain culture at a dose of 9 log CFU/mL experienced 100% mortality, whereas 8 log CFU/mL resulted in only 33% mortality.

**Fig. 3:**
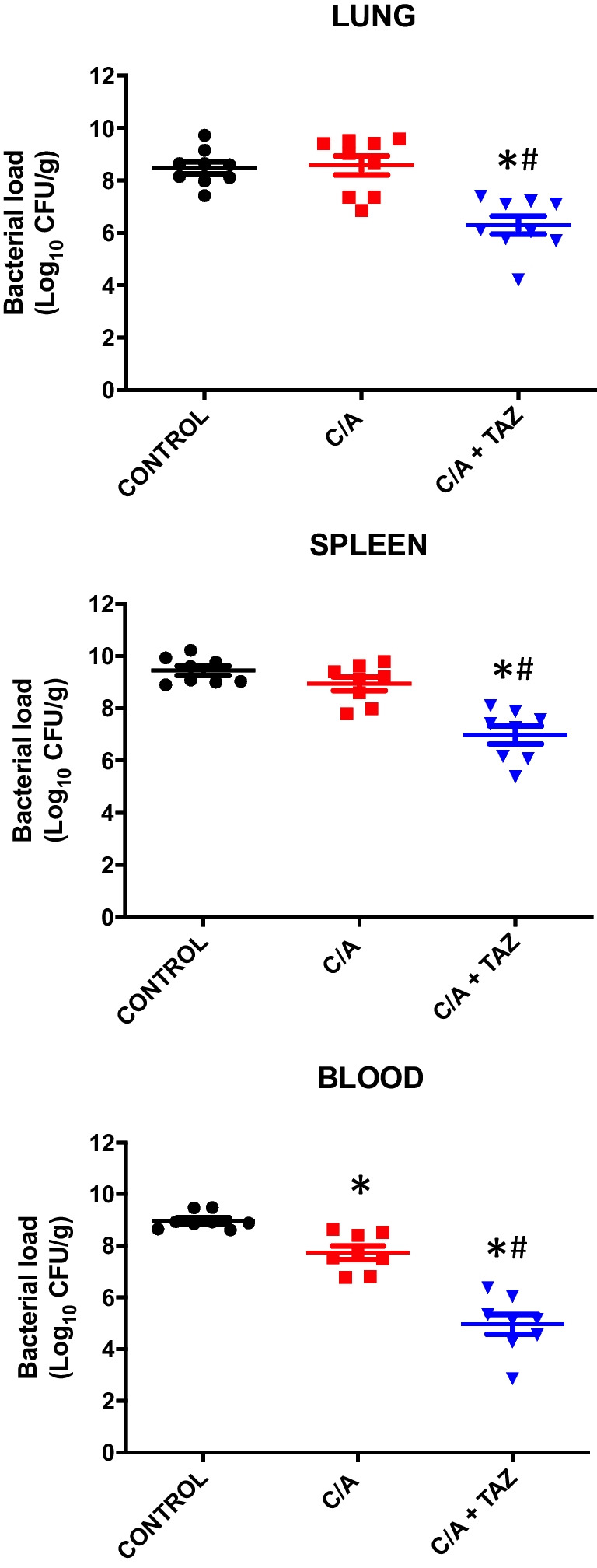
Therapeutic effect of clavulanic acid alone and in combination with tazobactam in vivo against carbapenem-resistant *A. baumannii*. Bacterial load in tissues and blood in the murine peritoneal sepsis model with 0.5 mL of *A. baumannii* Ab40 OXA-24 producer strain at 9 log CFU/mL. *P < 0.05 vs. control; ^#^*P* < 0.05 *vs*. the C/A treatment group. C/A, clavulanic acid; TAZ, tazobactam

In a murine model of peritoneal sepsis, C/A (13 mg/kg/4 h, i.p.) was combined with tazobactam (32 mg/kg/4 h, i.p.) and administered to mice 4 h after intraperitoneal exposure to the 0.5 mL of Ab40 strain culture at a dose of 9 log CFU/mL, which caused 100% mortality. The combination treatment was found to significantly reduce the bacterial load in the lung and spleen by 2.27 and 2.46 log_10_ CFU/g (*P* < 0.05) and in the blood by 4.01 log_10_ CFU/mL (*P* < 0.05), compared to control groups. In contrast, treatment with C/A alone did not significantly decrease the bacterial load in the lung and spleen relative to control groups. In blood, slight statistically significant decrease has been observed (Fig. 3).

## Discussion

The emergence of broad-spectrum antibiotic resistance in *A. baumannii* species has led to the search for new therapeutic alternatives. Due to the expression of resistance genes, bacteria have become resistant to BL antibiotics [28, 29], as well as to the combination of several BL-BLI [30–35]. This has prompted us to suggest that the combination of several BLIs could be an effective solution to this problem. In this study, we showed that the BLI C/A in combination with another BLI, tazobactam, acts synergistically against clinical isolates of *A. baumannii*. According to previous studies, none of both BLI have antibacterial activity in monotherapy against carbapenem-resistant *A. baumannii*. In this study, C/A alone had no activity against clinical isolates of *A. baumannii* and had a MIC range from 16 to >256 mg/L. The addition of avibactam did not increase the activity of C/A (data not shown). This result is in line with previous studies that showed that *Acinetobacter* spp. are largely resistant to ceftazidime-avibactam [32, 33]. In contrast, the combination of C/A with tazobactam in this study showed very promising results. The MICs for this combination range from <1 to >256 mg/L. It is noteworthy that the MIC_50_ of C/A in combination with tazobactam for the 24 analyzed carbapenem-intermediate and resistant strains decreased to 16 mg/L, 8-fold lower than the MIC_50_ of C/A alone. In next studies, these promising findings should be validated in large collections of clinical *A. baumannii* strains.

The results from microdilution assays were confirmed by checkerboard assays and time-kill curves. Combining 4 and 8 mg/L of tazobactam with 1xMIC of C/A showed synergy and bactericidal activity against Ab40, an OXA-24 producer strain, and Ab293, a lacking OXA β-lactamase strain. According to EUCAST guidelines, tazobactam is used with a fixed concentration of 4 mg/L for susceptibility testing. Our results suggest that increasing the concentration of tazobactam from 4 to 8 mg/L enhances the activity of C/A against clinical isolates of *A. baumannii*. This could be due to the similar spectrum of activity between C/A and tazobactam [3, 33].

The mode of action of the combination of C/A and tazobactam remains unknown. Like other β-lactams, such as sulbactam, whose mechanism of action is associated with binding to PBPs in *A. baumannii* [9], C/A has been demonstrated to bind to PBPs in *Escherichia coli* and other Gram-negative pathogens [36, 37]. However, there is currently no available information on tazobactam’s effect on PBPs. It is possible that the synergy observed in this study between C/A and tazobactam could be the result of a certain degree of additive effect in inhibiting PBPs.

Furthermore, with reference to the study by Fernandez et al. (2012), which assessed the expression of OXA-24 and OXA-10 in *E. coli*, it was demonstrated that the presence of these enzymes was linked to a low level of cross-linked peptidoglycan and longer sugar chains. This observation suggests that the expression of specific β-lactamases might be connected to alterations in the cell wall structure, potentially resulting in a diminished fitness both in vitro and in vivo [38]. This information could prove relevant for the consideration of future studies involving the Ab40 strain.

C/A is a broad-spectrum inhibitor that can inhibit most class A β-lactamases, including ESBLs and common TEM and SHV enzymes [3, 14]. Similarly, tazobactam has activity against many Ambler class A β-lactamases (TEM, SHV, and CTX-M-type) and some class C (AmpC-type) β-lactamases [14]. Both C/A and tazobactam inhibit most Ambler class A β-lactamases (excluding carbapenemases like KPC-2), but not those from Ambler classes B, C, or D [39].

On the other hand, our previous studies showed that *A. baumannii* relies adherence invasion to host cells as an initial and crucial step in causing infections [26, 40, 41]. However, no data have been reported on the combined effect of C/A and tazobactam on *A. baumannii*’s interaction with host cells. To our knowledge, this study provides the first evidence for the enhanced effect of tazobactam on C/A in reducing *A. baumannii*’s adherence invasion to host cells. Moreover, this effect is more pronounced against the Ab293 strain than the Ab40 strain, consistent with time-kill curve data indicating that C/A plus tazobactam is more bactericidal against Ab293, a lacking OXA β-lactamase strain, than Ab40, an OXA-24 producer strain.

Animal infection models are useful for studying the potential uses of β-lactamase inhibitors both as monotherapy and in combination. For example, the efficacy of ETX2514 in combination with sulbactam reduced the bacterial loads more than monotherapy with ETX1514 or sulbactam in neutropenic mouse thigh infection model by carbapenem-resistant *A. baumannii* [21]. In this study, the combination of C/A and tazobactam was effective against carbapenem-resistant *A. baumannii* in a murine peritoneal sepsis model. The combination reduced the bacterial load by around 2.5 log_10_ CFU/g in tissues and 4 log_10_ CFU/mL in blood, while there was no significant difference between C/A monotherapy and the control group. Tazobactam has been also showed to enhance the activity of colistin in murine pneumoniae model caused by a virulent *A. baumannii* strain [27].

The animal model of this study was not used beyond 24 h because of the high number of C/A doses that had to be administered to the animals to reach a serum concentration above the MIC for at least 40% of the time between doses [14]. In spite of this, combination treatment showed significant decrease in bacterial loads. The in vivo data shown do not exhibit a significant correlation with host survival. Naturally, numerous other factors come into play here that could lead to mortality, such as the excessive stimulation and promotion of proinflammatory cytokines like IL-6 and TNF-alpha [42, 43]. Nevertheless, the results are intriguing and offer a noteworthy strategy to enhance the effectiveness of existing β-lactams, while not excluding the consideration of other adjuvants or alternative therapies concurrently. Furthermore, this study possesses certain limitations due to the relatively low number of tested strains. We are of the opinion that the next focal point should involve expanding the bacterial collections to corroborate the therapeutic efficacy of the C/A combination with tazobactam.

## Conclusions

This study provides new insights on the use of BLIs against carbapenem-resistant *A. baumannii* clinical isolates. Exploring novel combinations may offer new options to treat *A. baumannii* infections, which have limited treatment options.

## Data Availability

The data that support the findings of this study are available from the corresponding author upon reasonable request.
